# Comparative validation and local adaptation of gastric cancer risk assessment models in a large multicenter endoscopic screening cohort in China

**DOI:** 10.1186/s12916-026-05082-6

**Published:** 2026-07-24

**Authors:** Xue Li, Ying Chen, Juan Zhu, Wei-Yan Yu, Huan-Qing Tao, Gang Chen, Hui-Zhang Li, Ling-Bin Du

**Affiliations:** 1 Department of Cancer Prevention, Zhejiang Cancer Hospital, Hangzhou Institute of Medicine (HIM), Chinese Academy of Sciences, Hangzhou, 310022 Zhejiang China; 2 Department of Epidemiology, School of Public Health, Nanjing Medical University, Nanjing, 211166 Jiangsu China

**Keywords:** Gastric cancer, Cancer screening, Cancer prevention, Risk assessment, Model construction

## Abstract

**Background:**

Gastric cancer (GC) is a major public health challenge in China, highlighting the need for effective risk stratification to improve early detection. This study aimed to validate existing GC risk assessment models and develop a locally adapted screening approach for GC and precancerous lesions.

**Methods:**

From 2022 to 2024, a total of 144,329 participants were recruited through a large multicenter endoscopic screening cohort in Zhejiang Province, China, with baseline data collected via interviews and endoscopic examinations. Participants screened in 2022 were used for model development, whereas those screened during 2023–2024 were used for validation and comparative analyses. Least absolute shrinkage and selection operator regression and logistic regression were used to construct a new model and a weighted point-based risk score. Six previously published models were externally validated for comparison across the validation set. Model performance and clinical utility were assessed and compared using area under the curve (AUC), detection rates, and decision curve analysis for both GC and the composite outcome of GC/high-grade intraepithelial neoplasia (HGIN).

**Results:**

A total of 1,419 GC cases were identified in the screening cohort. The newly developed model demonstrated favorable discriminative performance, with an AUC of 0.759 (95% CI: 0.744–0.775) for GC and 0.754 (95% CI: 0.740–0.768) for the composite outcome of GC/HGIN in the validation set. External evaluation of six previously published models showed AUCs ranging from 0.594 (95% CI: 0.558–0.629) to 0.742 (95% CI: 0.725–0.758) for GC, and from 0.602 (95% CI: 0.570–0.635) to 0.744 (95% CI: 0.730–0.759) for GC/HGIN. Risk stratification analyses demonstrated progressively increasing odds of both GC and GC/HGIN across higher-risk quartiles in most evaluated models. In the newly developed model, the highest-risk quartile accounted for 62.25% of GC cases and 58.56% of HGIN cases, indicating substantial enrichment of clinically significant lesions. Decision curve analysis indicated a favorable net benefit of the newly developed model across the evaluated range of threshold probabilities.

**Conclusions:**

The newly developed model demonstrated favorable discrimination, stable geographic performance, and effective risk stratification in a large multicenter screening cohort. These findings support its potential utility for risk-adapted GC screening in Chinese populations.

**Graphical abstract:**

**Figure:**
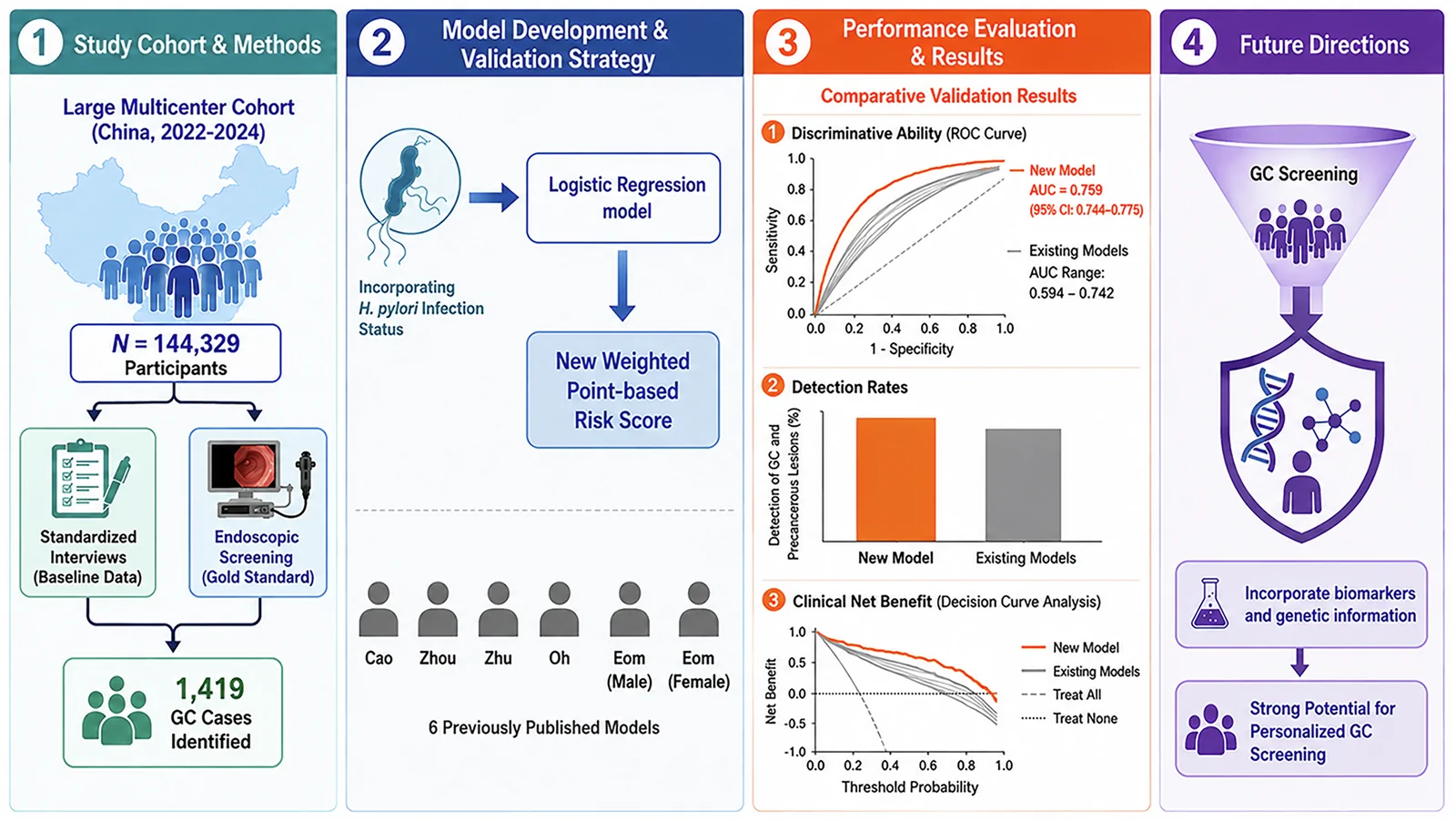

## Background

Gastric cancer (GC) is a leading gastrointestinal malignancy worldwide. According to GLOBOCAN 2022, there were approximately 968,000 new GC cases and 660,000 deaths globally in 2022, accounting for 4.9% of total cancer incidence and 6.8% of cancer-related mortality, ranking fifth in both cancer incidence and mortality [1]. China bears a disproportionately high burden of GC, reporting 260,400 GC-related deaths in 2022 [2]. Recent estimates indicate that new GC cases in China account for approximately 44% of the global burden, representing the highest number of GC cases worldwide [3]. Although clinical treatments have advanced, a substantial proportion of patients are still diagnosed at an advanced stage, complicating treatment and worsening prognosis [4]. Thus, early screening, diagnosis and treatment of GC are critical.

Endoscopy is widely regarded as the gold standard for GC detection. Previous studies have consistently demonstrated that endoscopic screening and early diagnosis significantly reduce both GC incidence and mortality [4–6], with repeated endoscopic surveillance further enhancing screening effectiveness [7]. However, the relatively low prevalence of GC and precancerous lesions in the general population raises concerns about the efficiency of universal endoscopic screening, as it may lead to unnecessary procedures and associated physical and psychological burdens for low-risk individuals. In China, additional challenges such as high screening costs, uneven distribution of medical resources, and limited patient compliance further hinder the implementation of mass endoscopic screening programs.

To improve screening efficiency and enable risk-stratified approaches, there has been growing interest in the use of risk assessment models as a non-invasive, preliminary screening tool. These models typically incorporate epidemiological characteristics, lifestyle factors, and medical history to generate individualized risk scores and stratify patients into different risk categories. This approach facilitates tailored screening interventions and optimizes the allocation of endoscopic resources.

Although several GC risk assessment models have been proposed in recent years, most were derived in specific or restricted populations. Their generalizability, stability, and clinical applicability across diverse regions and large-scale populations have not been sufficiently validated. Moreover, the development of a locally adapted model suitable for GC screening is an urgent need.

To address these gaps, this study utilized data from a large-scale, multicenter endoscopic screening cohort in Zhejiang Province, China (2022–2024), with the following objectives: (1) to establish a new risk assessment model tailored to the local population; (2) to comparatively validate previously published GC risk assessment models in a temporally independent validation cohort; and (3) to compare the new model with existing models in terms of discriminatory ability, risk stratification capability, and clinical utility. The evidence generated from this study may provide a practical tool for GC risk assessment in China and support the development of risk-stratified screening strategies to improve the efficiency and effectiveness of GC prevention.

## Methods

### Study design and population

This study was conducted based on the data from an endoscopic screening cohort in Zhejiang Province, China (2022–2024). The study consisted of two parts: the first part aimed to develop a risk assessment model for GC, and the second part aimed to comparatively validate previously published GC risk assessment models. Participants screened in 2022 were assigned to the training set for model development, whereas participants screened during 2023–2024 were assigned to the validation set for model validation and model comparison analyses. To ensure a fair comparison and avoid potential bias related to model development, all external models and the newly developed model were evaluated exclusively within the validation set, which was independent from the training dataset used for new model construction.

**Fig. 1:**
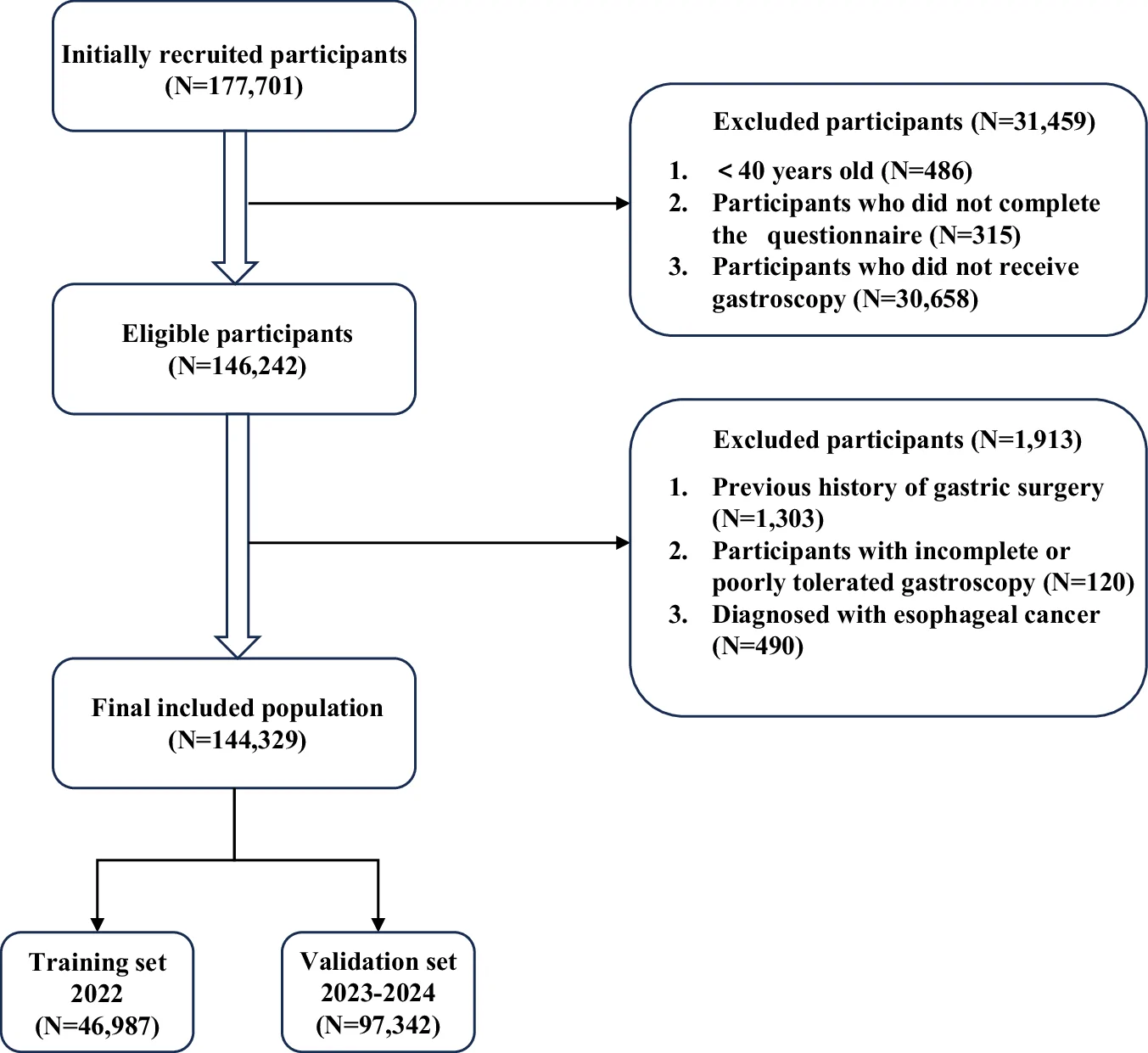
Flowchart of participants selection, model development, and validation

For the development of the new gastric cancer risk assessment model, participants were eligible if they met the following inclusion criteria: (1) registered permanent residents of Zhejiang Province or residents who had lived in the province for at least three years; (2) aged 40–74 years old; (3) voluntarily participated and provided written informed consent. Exclusion criteria included: (1) severe cardiopulmonary disease (2) other conditions preventing toleration of endoscopy; and (3) poor cooperation during endoscopy. During 2022–2024, a total of 177,701 participants were recruited. After excluding individuals younger than 40 years (n = 486) and those with incomplete questionnaire data (n = 315), 176,900 participants remained, among whom 146,242 underwent gastroscopy. Further exclusions were applied for prior gastric surgery (n = 1,303), incomplete or poorly tolerated gastroscopy (n = 120), and newly diagnosed esophageal cancer (n = 490), resulting in a final analytic cohort of 144,329 participants, and 46,987 participants from 2022 and 97,342 participants from 2023–2024 were assigned to the training set and validation set respectively (Fig. 1).

### Data collection and variables

Questionnaire data were collected through face-to-face interview. The survey included the following categories: (1) demographic characteristics, including age, sex, educational level, and date of birth; (2) physical examination data, including height, weight, and waist circumference, with BMI calculated as weight (kg) divided by height squared (m²); (3) lifestyle factors, including smoking (defined as ≥1 cigarette/day for ≥6 consecutive or cumulative months), alcohol consumption (defined as ≥1 drinking episode/week for ≥6 consecutive or cumulative months), preference for hot beverages and foods, frequency of intake of pickled foods, fresh vegetables, and fresh fruits; (4) medical history, including upper gastrointestinal symptoms (nausea, vomiting, dyspepsia, bloating, acid reflux, belching, etc.), history of gastrointestinal ulcers, prior gastroscopy, first-degree family history of cancer, and history of gastrointestinal diseases (low-grade intraepithelial neoplasia of the esophagus, Barrett’s esophagus, gastrointestinal metaplasia, low-grade gastric intraepithelial neoplasia, atrophic gastritis, chronic gastritis, duodenal ulcer, or residual stomach after benign surgery); and (5) *Helicobacter pylori* (*H. pylori*) infection status, with all participants tested using urea breath test, urease test, stool or serological assays, prioritizing the breath test when feasible.

Questionnaire administration was conducted by trained research personnel who ensured that participants fully understood each question and provided accurate responses while maintaining privacy. Personal medical history and family history of cancer were based on self-report.

### Endoscopic examination and definition of outcomes

Endoscopic examinations were performed according to standardized clinical practice, and national gastric cancer screening protocols [8]. Examination results were recorded in the cancer screening information system using unified and detailed reporting forms for both endoscopic and pathological findings. All suspected lesions identified during endoscopy were subjected to biopsy and histopathological examination, with the number of biopsy specimens determined by lesion size, morphology, and distribution. In cases of multiple lesions, the final diagnosis was based on the lesion with the highest pathological grade.

Biopsy processing and pathological diagnoses followed internationally accepted criteria, including the WHO Classification of Tumours of the Digestive System and the American Joint Committee on Cancer (AJCC) staging system (8th edition). To ensure diagnostic consistency and quality control, Zhejiang Cancer Hospital regularly organized standardized cancer screening training programs for physicians from participating screening centers twice annually, covering standardized endoscopic and pathological diagnostic procedures. Each pathological diagnosis was independently evaluated by two experienced gastrointestinal pathologists. In cases of discordance, a third senior pathologist reviewed the specimen or a consensus diagnosis was reached through joint discussion. In addition, a formal central review committee led by Zhejiang Cancer Hospital was established for this study. All gastric precancerous lesions (GPL) and gastric cancer cases underwent final review and diagnostic confirmation by experienced endoscopists and pathologists within the committee.

Outcomes were classified into five categories, including pathologically confirmed gastric cancer (including cardia cancer and gastric nonepithelial tumors, e.g., neuroendocrine carcinoma, neuroendocrine tumor, lymphoma, and mesenchymal-origin tumors), gastric precancerous lesions (including low-grade intraepithelial neoplasia [LGIN] and high-grade intraepithelial neoplasia [HGIN]), precancerous conditions (intestinal metaplasia, atrophic gastritis), inflammation/normal mucosa, and other pathological diagnoses (such as fundic gland polyps and hyperplastic polyps).

### Development of a new GC assessment model

Study data were chronologically divided into a training set (participants enrolled in 2022) and a validation set (participants enrolled in 2023–2024).

Model development was conducted in three steps. First, univariate logistic regression analyses were performed to evaluate the association between each baseline variable and gastric cancer incidence, estimating odds ratios (ORs) and corresponding 95% confidence intervals (CIs). Variables with statistical significance in the univariate analyses (*P* < 0.05) were subsequently included in a least absolute shrinkage and selection operator (LASSO) regression model for variable selection, with lambda.1se adopted as the selection criterion. Variables retained by the LASSO regression were then entered into a multivariable logistic regression model to construct the final risk assessment model. Subsequently, a risk scoring system was constructed based on the regression coefficients derived from the multivariable logistic regression model. Among the statistically significant predictors, the variable with the smallest absolute regression coefficient (β) was assigned a risk score of 1.0 point. The scores for the remaining variables were calculated according to their relative weights compared with the smallest absolute β coefficient. Individual risk scores were obtained by summing the assigned scores across all predictors.

Model performance was assessed using receiver operating characteristic (ROC) curves and area under the curve (AUC) values in both the training and validation sets. To further examine the geographic robustness of the model across Zhejiang Province, the validation cohort was additionally stratified into three geographic regions: Center (Hangzhou, Jiaxing, Shaoxing, and Huzhou), East (Ningbo, Wenzhou, Taizhou, and Zhoushan), and Southwest (Jinhua, Lishui, and Quzhou). ROC curves and AUCs were evaluated separately within each region subset.

### Models selection from literature

A targeted literature screening approach was employed to identify gastric cancer risk assessment models from peer-reviewed articles published in authoritative journals for comparative validation. The inclusion criteria for model selection were as follows: (1) study populations with demographic characteristics (e.g., age distribution, geographic region, ethnicity) comparable to those of the present study; (2) inclusion of variables identical or similar to those collected in this study’s questionnaire; (3) provision of a reproducible risk score calculation formula or defined scoring criteria; and (4) original research published in English. Exclusion criteria included: (1) models not designed for gastric cancer risk assessment (e.g., prognostic models); (2) models with outcomes limited to specific subtypes of gastric cancer; (3) models targeting populations substantially different from the current study population (e.g., adolescents); (4) models with excessive missing variables or major discrepancies in the variable definitions compared with the current questionnaire dataset; and (5) models incorporating serological tests or biomarkers (e.g., pepsinogen, gastrin, or omics biomarkers). Biomarker-based models were excluded because several biomarkers were not uniformly available in the screening dataset, and the present study aimed to focus on models based on readily obtainable demographic and clinical questionnaire variables that are more feasible for large-scale population-based screening.

A systematic search of PubMed was conducted using the keywords “gastric cancer,” “stomach cancer,” “prediction model,” and “risk” to identify relevant articles published up to August 2025. Based on the inclusion and exclusion criteria, five eligible studies were selected. Five studies providing six distinct models [9], Eom (Female) [9], Oh [10], Cao [11], Zhou [12], Zhu [13]] were included. A structured data extraction form was used to record key information for each model, including first author, year of publication, country, participant age, study design, model type, sample size, included risk factors, discrimination power (Additional file: Table S1). To address discrepancies in variable definitions, the research team established a standardized variable harmonization protocol to ensure that the variable definitions were consistent with those in the original model. If a variable’s categorization or definition in a published model was inconsistent with the questionnaire used in this study, it was recategorized based on its conceptual meaning, or replaced with a variable of similar definition (Additional file: Table S2). All variables included in the models and their calculation methods were independently completed by two researchers (Y. C. and W-Y. Y.), and any discrepancies were resolved through team discussion to reach a consensus.

### External validation and comparison of models

Using data from the validation set, individual risk scores were calculated for each participant according to the original variable definitions and scoring algorithms of the six selected gastric cancer risk assessment models and the newly developed model. Differences in risk scores between participants with and without gastric cancer were subsequently compared. Because the required variables, eligibility criteria and applicable age ranges differed across previously published models, validation analyses were conducted within model-specific applicable subsets of the validation set. For the sex-specific models developed by Eom et al., validation analyses were conducted separately within the corresponding eligible male and female subpopulations of the validation set. Accordingly, the sample size varied slightly across different model validation analyses. For risk stratification analyses, participants were categorized into four risk groups (Q1-Q4) according to the quartile distribution of model-specific risk scores within each applicable study population. For sex-specific models, quartiles were also determined separately within the eligible male and female subpopulations. Detection rates of gastric cancer and other gastric lesions, including precancerous lesions (LGIN and HGIN), precancerous conditions, inflammation/normal, and other pathological diagnoses), were compared across the risk groups to evaluate each model’s ability to identify high-risk individuals. Because the previously published comparator models were originally developed for GC risk assessment, GC was retained as the primary outcome for model validation and comparison analyses. Considering that HGIN is clinically managed similarly to early GC and often requires definitive endoscopic treatment, a secondary analysis was performed using a composite outcome of GC and HGIN. For predictive value analyses, Q4 was defined as the high-risk (test-positive) group and Q1-Q3 as the non-high-risk (test-negative) group. Positive predictive value (PPV) was calculated as the proportion of participants with the specified outcome in Q4, whereas negative predictive value (NPV) was calculated as the proportion of participants without the specified outcome in Q1-Q3. Odds ratios for Q2-Q4 were calculated using Q1 as the reference group for the primary outcome of GC and the secondary outcome of GC/HGIN. Receiver operating characteristic curves, area under the curve, and decision curve analysis (DCA) were used to evaluate and compare model discrimination and clinical utility for both GC and GC/HGIN outcomes.

### Statistical analysis

All data cleaning, management, and statistical analyses were conducted using R (version 4.4.2). Baseline population characteristics were summarized using descriptive statistics: continuous variables with a normal or approximately normal distribution were expressed as mean ± standard deviation (mean ± SD), and categorical variables were presented as frequencies and percentages (n, %). Differences in categorical variables between groups were assessed using the chi-square (*χ²*) test. Logistic regression analysis was employed for the development of the new model. Differences in AUCs between the new model and other models were compared using Bootstrap test. All statistical tests were two-sided, and *P* < 0.05 was considered statistically significant.

## Results

### Baseline characteristics

The model construction and validation were based on the endoscopic screening cohort in Zhejiang Province, including a total of 144,329 participants with a mean age of 56.71 ± 8.51 years. Of these, 67,870 (47.02%) were male and 76,459 (52.98%) were female. A total of 1,419 GC cases (0.98%) and 142,910 non-GC cases (99.02%) were identified. Regarding endoscopic and pathological results, 94,637 (65.57%) participants had inflammation/normal findings, 42,972 (29.77%) participants had precancerous conditions, and 1,792 (1.24%) participants had precancerous lesions, including 1,460 LGIN cases (1.01%) and 332 HGIN cases (0.23%). In addition, 3,509 (2.43%) participants had other pathological diagnoses. Comparative analysis showed that, except for history of peptic ulcer disease and family history of GC in first-degree relatives (*P* > 0.05), all other factors differed significantly between GC and non-GC groups (*P* < 0.05) (Table 1).

**Table 1:** Baseline characteristics of the participants in the endoscopic screening cohort

Characteristic	All (n = 144,329)	Non-gastric cancer (n = 142,910)	Gastric cancer (n = 1,419)	*χ*^2^ value	*P* value
Age (years) (n, %)				881.60	<0.001
40–49	31,709 (21.97%)	31,613 (22.12%)	96 (6.77%)		
50–59	58,167 (40.30%)	57,820 (40.46%)	347 (24.45%)		
60–69	43,734 (30.30%)	43,102 (30.16%)	632 (44.54%)		
70–74	10,719 (7.43%)	10,375 (7.26%)	344 (24.24%)		
Sex (n, %)				287.50	<0.001
Female	76,459 (52.98%)	76,025 (53.20%)	434 (30.58%)		
Male	67,870 (47.02%)	66,885 (46.80%)	985 (69.42%)		
Education (n, %)				194.00	<0.001
Primary school education or below	60,030 (41.59%)	59,211 (41.43%)	819 (57.72%)		
Junior high school	47,693 (33.04%)	47,273 (33.08%)	420 (29.60%)		
Senior high school	21,556 (14.94%)	21,423 (14.99%)	133 (9.37%)		
Junior college education or above	15,050 (10.43%)	15,003 (10.50%)	47 (3.31%)		
BMI, kg/m^2^ (n, %)				33.9	<0.001
<18.5	3,671 (2.54%)	3,600 (2.52%)	71 (5.00%)		
≥18.5	140,658 (97.46%)	139,310 (97.48%)	1,348 (95.00%)		
Smoking (n, %)				192.80	<0.001
No	119,349 (82.69%)	118,373 (82.83%)	976 (68.78%)		
Yes	24,980 (17.31%)	24,537 (17.17%)	443 (31.22%)		
Drinking (n, %)				121.93	<0.001
No	121,259 (84.02%)	120,219 (84.12%)	1,040 (73.29%)		
Yes	23,070 (15.98%)	22,691 (15.88%)	379 (26.71%)		
Intake of pickled food (n, %)				85.67	<0.001
≤3 days/week	119,881 (83.06%)	118,833 (83.15%)	1,048 (73.85%)		
>3 days/week	24,448 (16.94%)	24,077 (16.85%)	371 (26.15%)		
Intake of vegetables (n, %)				26.51	<0.001
≤3 days/week	23,066 (15.98%)	22,768 (15.93%)	298 (21.00%)		
>3 days/week	121,263 (84.02%)	120,142 (84.07%)	1,121 (79.00%)		
Intake of fruits (n, %)				93.47	<0.001
≤3 days/week	60,487 (41.91%)	59,713 (41.78%)	774 (54.55%)		
>3 days/week	83,842 (58.09%)	83,197 (58.22%)	645 (45.45%)		
Intake of hot food/tea (n, %)				57.79	<0.001
No	115,665 (80.14%)	114,642 (80.22%)	1,023 (72.09%)		
Yes	28,664 (19.86%)	28,268 (19.78%)	396 (27.91%)		
History of gastroscopy (n, %)				155.26	<0.001
No	105,639 (73.19%)	104,393 (73.05%)	1,246 (87.81%)		
Yes	38,690 (26.81%)	38,517 (26.95%)	173 (12.19%)		
History of peptic ulcer (n, %)				0.01	0.905
No	138,903 (96.24%)	137,536 (96.24%)	1,367 (96.34%)		
Yes	5,426 (3.76%)	5,374 (3.76%)	52 (3.66%)		
Upper gastrointestinal symptoms (n, %)				209.37	<0.001
No	81,706 (56.61%)	81,172 (56.80%)	534 (37.63%)		
Yes	62,623 (43.39%)	61,738 (43.20%)	885 (62.37%)		
Family history of gastric cancer in first-degree relatives (n, %)				0.44	0.507
No	142,051 (98.42%)	140,658 (98.42%)	1,393 (98.17%)		
Yes	2,278 (1.58%)	2,252 (1.58%)	26 (1.83%)		
*H. pylori* infection status (n, %)				84.92	<0.001
Positive	39,875 (27.63%)	39,328 (27.52%)	547 (38.55%)		
Negative	104,454 (72.37%)	103,582 (72.48%)	872 (61.45%)		

*BMI* body mass index, *H. pylori Helicobacter pylori*

Participants were divided into a training set and a validation set according to the year of screening for the development and validation of the new GC risk assessment model. The training set comprised 46,987 individuals (484 GC cases and 46,503 non-GC cases) screened in 2022, with a GC detection rate of 1.03%. The validation set included 97,342 individuals (935 GC cases and 96,407 non-GC cases) screened in 2023–2024, with a GC detection rate of 0.96%. Baseline characteristics of participants in the training and validation sets are shown in Additional file Table S3.

### Development and validation of the new model

In the training set, univariate logistic regression analysis was first performed to examine the associations between candidate variables and GC risk. 13 variables were found to be significantly associated with GC occurrence (*P* < 0.05, Additional file: Table S4). After LASSO regression selection, 9 variables were retained for the final model construction, including age, sex, education, smoking, upper gastrointestinal symptoms, history of gastroscopy, intake of vegetables, intake of fruits, and *H. pylori* infection status. Risk scores for each variable were derived from the regression coefficients of the final model, with the variable having the smallest absolute regression coefficient (smoking, β = 0.248, *P* = 0.029) assigned a score of 1.0 point as the reference. Scores for the remaining variables were calculated according to their relative weights compared with the smoking coefficient. The individualized risk score was obtained by summing the scores of all predictors and ranged from 0 to 25.5 points, with higher scores indicating a greater likelihood of GC occurrence (Table 2).

**Table 2:** Multivariable logistic regression analysis and corresponding risk scores for gastric cancer risk factors

Characteristic	*β* value	OR	95% CI	*P* value	Risk score
Age (years)					
40–49	Ref				0.0
50–59	0.439	1.55	1.07–2.25	0.02	1.8
60–69	1.305	3.69	2.57–5.29	<0.001	5.3
70–74	1.806	6.09	4.08–9.08	<0.001	7.3
Sex					
Female	Ref				0.0
Male	0.747	2.11	1.70–2.62	<0.001	3.0
Education					
Junior college education or above	Ref				0.0
Senior high school	0.604	1.83	1.00–3.33	0.048	2.4
Junior high school	0.692	2.00	1.14–3.50	0.016	2.8
Primary school education or below	0.836	2.31	1.32–4.03	0.003	3.4
Smoking					
No	Ref				0.0
Yes	0.248	1.28	1.03–1.60	0.029	1.0
Upper gastrointestinal symptoms					
No	Ref				0.0
Yes	0.536	1.71	1.41–2.07	<0.001	2.2
History of gastroscopy					
Yes	Ref				0.0
No	1.166	3.21	2.38–4.33	<0.001	4.7
Intake of vegetables					
>3 days/week	Ref				0.0
≤3 days/week	0.340	1.40	1.13–1.75	0.003	1.4
Intake of fruits					
>3 days/week	Ref				0.0
≤3 days/week	0.281	1.32	1.08–1.62	0.007	1.1
*H. pylori* infection status					
Negative	Ref				0.0
Positive	0.346	1.41	1.17–1.71	<0.001	1.4

*CI* confidence interval, *H. pylori* *Helicobacter pylori*, *OR* odds ratio

**Fig. 2:**
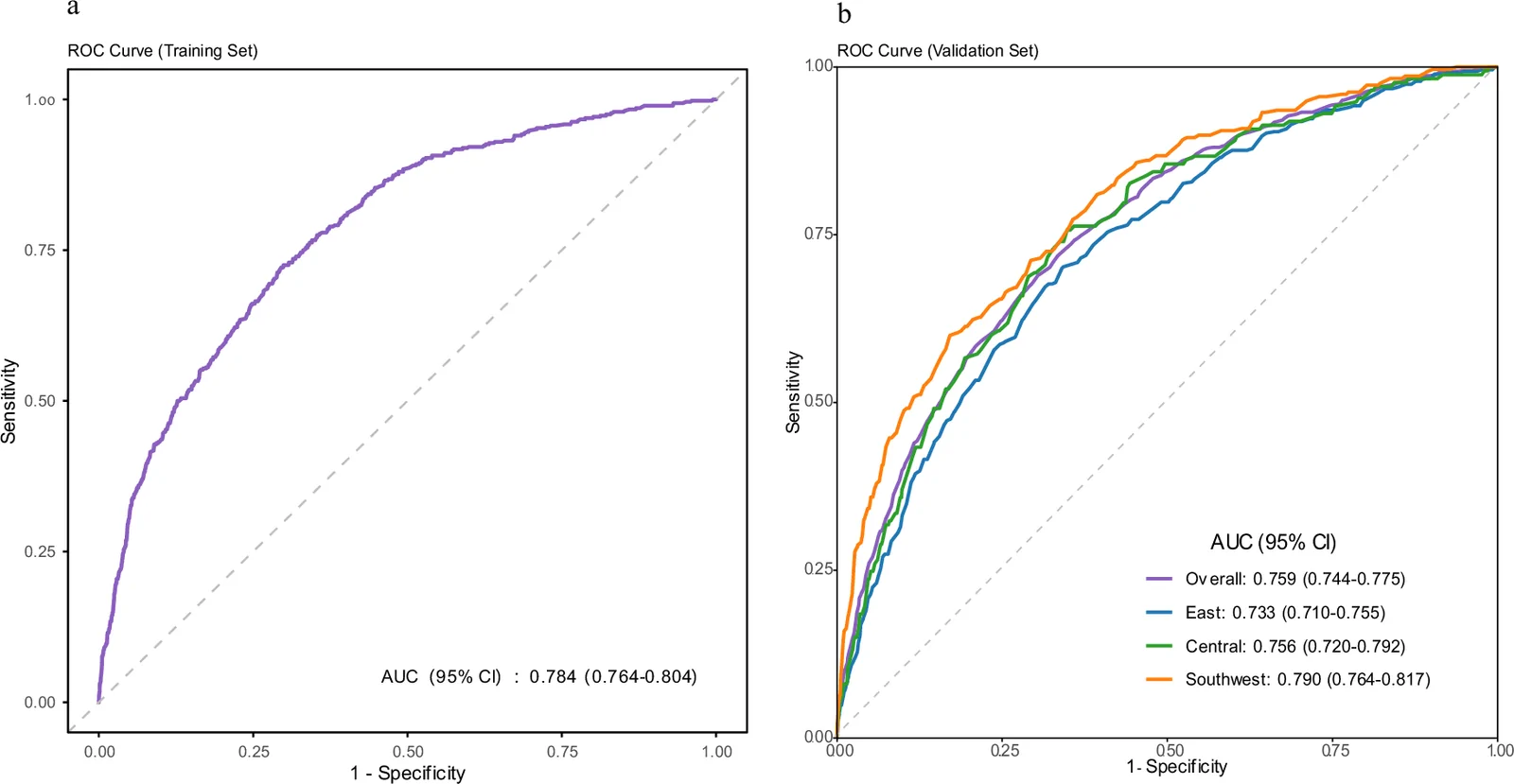
Receiver operating characteristic (ROC) curves of the newly developed model in the training and validation sets. **a**: Training set; **b**: Validation set and geographic subgroup validation in eastern, central, and southwestern Zhejiang Province

Applying the model to discriminate GC cases, the AUC was 0.784 (95% CI: 0.764–0.804) (Fig. 2a) in the training set and 0.759 (95% CI: 0.744–0.775) (Fig. 2b) in the validation set. The model demonstrated relatively consistent discriminatory performance across the three geographic regions of the validation set. The AUC values were 0.756(95% CI: 0.720–0.792) for the Center region, 0.733 (95% CI: 0.710–0.755) for the East region, and 0.790 (95% CI: 0.764–0.817) for the Southwest region (Fig. 2b).

### Model selection and characteristics of included models

A total of six previously published GC risk assessment models derived from Korean and Chinese populations were included in this study, namely the Eom (Male) [9], Eom (Female) [9], Oh [10], Cao [11], Zhou [12], and Zhu [13] models. These models varied in terms of applicable age ranges, modeling strategies, included variables, and sample sizes. In general, the models incorporated conventional epidemiological factors, including demographic characteristics (e.g., age, sex, BMI, family history), lifestyle factors (e.g., smoking, alcohol consumption, dietary habits), and medical history (e.g., history of endoscopy, history of gastric disease). The discrimination of the models was evaluated using the AUC (95% CI), which ranged from 0.706 (0.698–0.715) to 0.800 (0.770–0.829) in the development set, and from 0.696 (0.567–0.825) to 0.799 (0.743–0.856) in the validation set (Additional file: Table S1).

### Discrimination and risk stratification of the published models and the new model

**Fig. 3:**
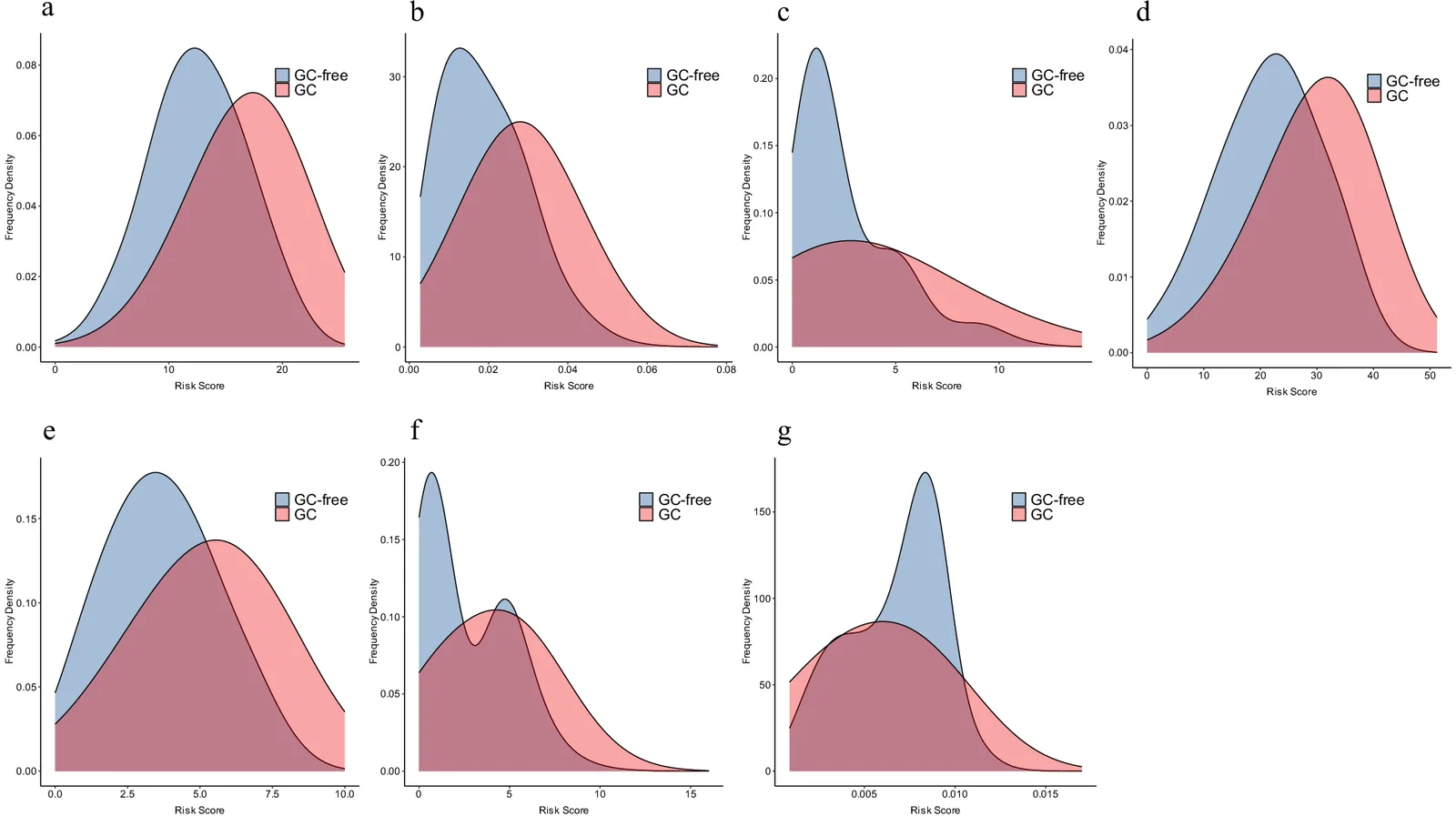
Distribution of risk scores between GC and non-GC participants across different models. **a**: New model **b**: Eom (Male) **c**: Oh **d**: Zhu **e**: Zhou **f**: Cao **g**: Eom (Female)

Six previously published GC risk assessment models and the newly developed model were validated and compared in the validation set. For the new model, Eom (Male) [9], Oh [10], Zhu [13], Zhou [12], and Cao [11], participants with GC had significantly higher risk scores than those of non-GC participants (all *P* < 0.001, Fig. 3a–f), whereas the Eom (Female) model [9] showed the opposite pattern (*P* < 0.001, Fig. 3g).

**Fig. 4:**
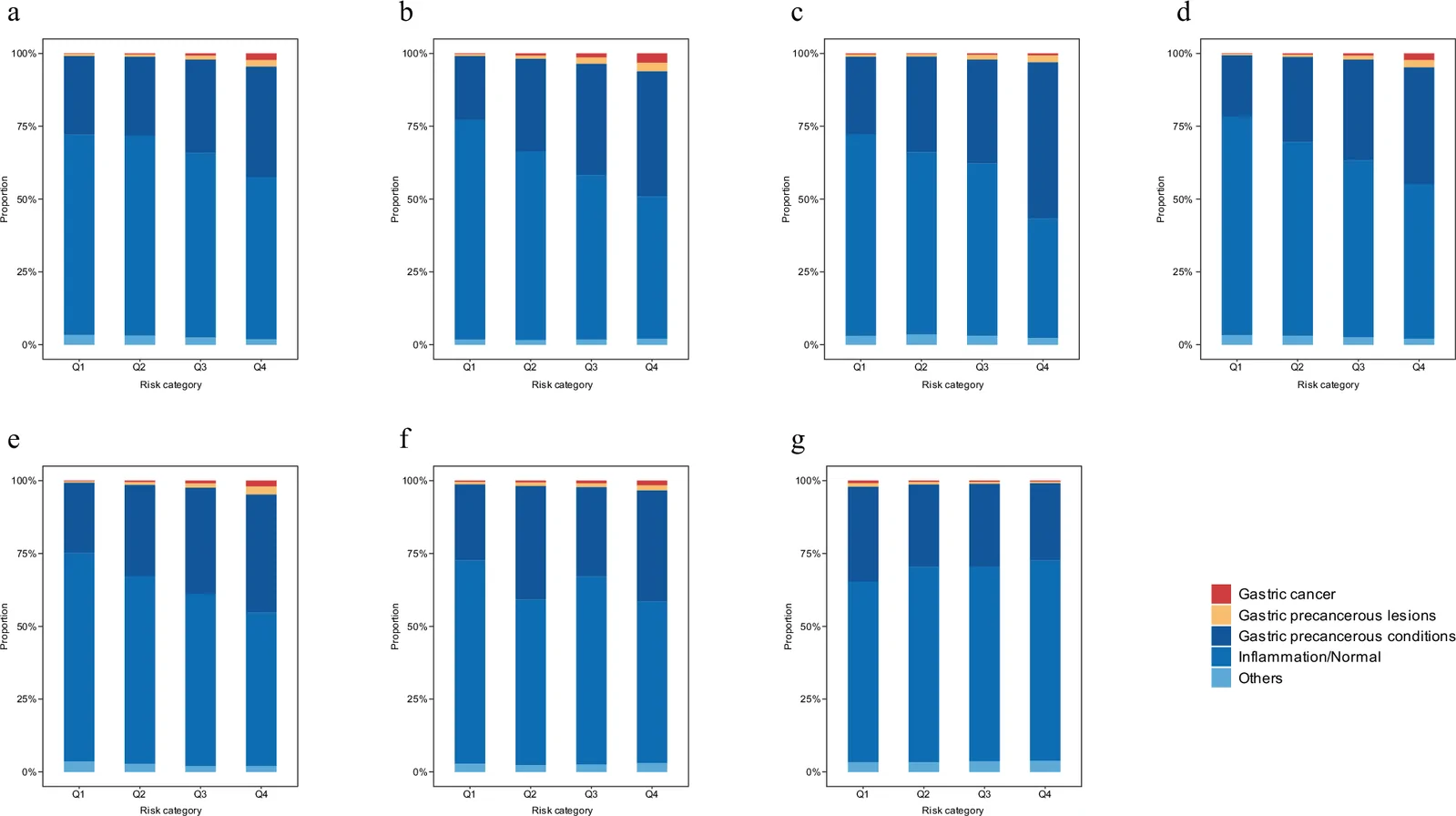
Distribution of gastric lesions across risk score quartiles for different models. **a**: New model **b**: Eom (Male) **c**: Oh **d**: Zhu **e**: Zhou **f**: Cao **g**: Eom (Female)

For outcome detection, the new model, Eom (Male) [9], Oh [10], Zhu [13], and Zhou [12] models demonstrated increasing detection rates of GC, GPL, and precancerous conditions across risk score quartiles, with the highest yields consistently observed in Q4 (Fig. 4a–e). The Cao model [11] showed a similar trend for GC and GPL (Fig. 4f). In contrast, the Eom (Female) model [9] showed an inverse distribution pattern (Fig. 4g).

**Fig. 5:**
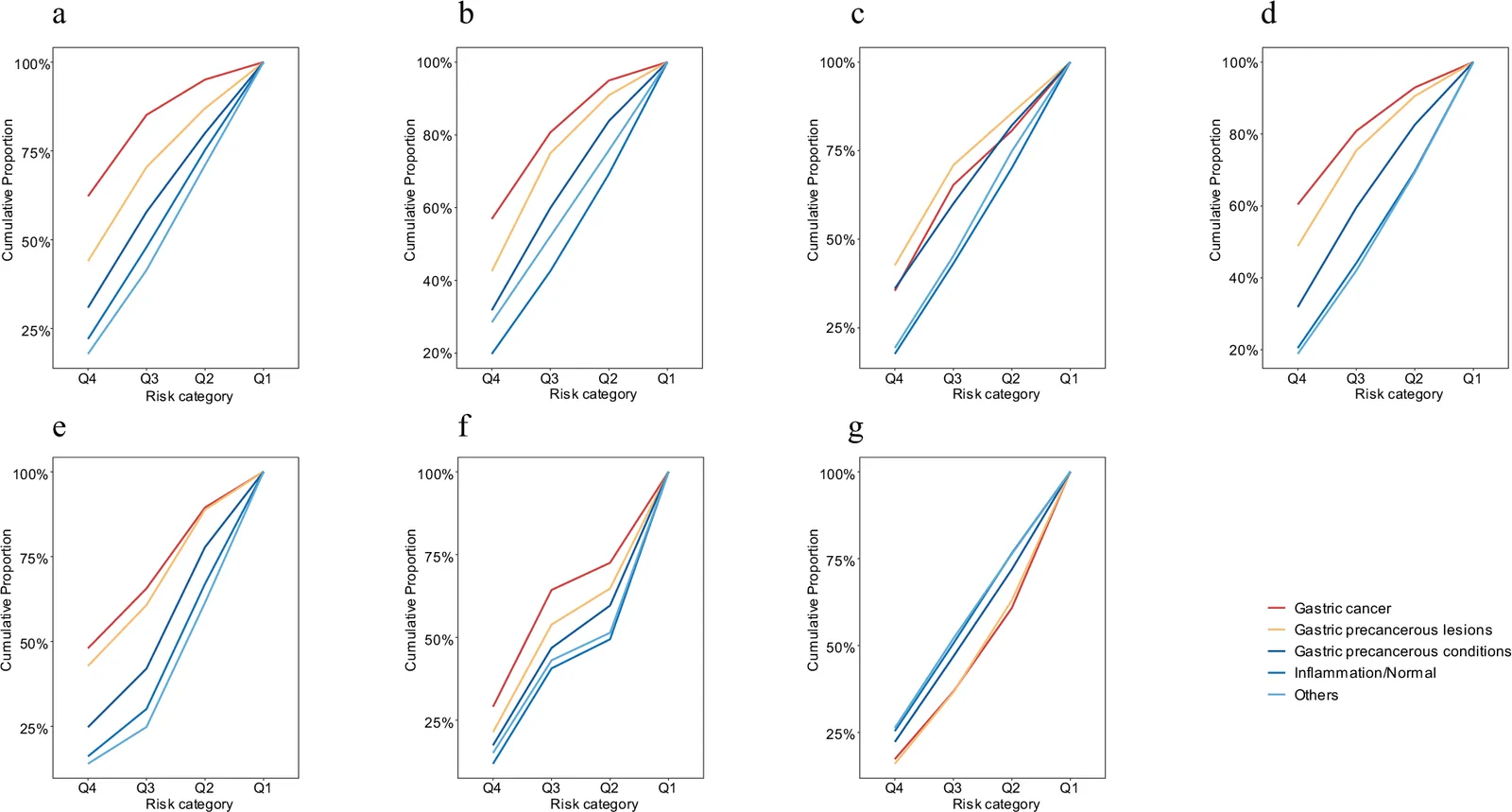
Cumulative contribution of different risk groups to the detection of gastric lesions across models. **a**: New model **b**: Eom (Male) **c**: Oh **d**: Zhu **e**: Zhou **f**: Cao **g**: Eom (Female)

The newly developed model stratified participants into four risk groups based on the quartiles of the risk scores, with Q4 defined as the high-risk group. Within Q4 582 GC cases, 540 GPL cases (410 LGIN cases and 130 HGIN cases), and 9,358 precancerous conditions were identified. The corresponding detection rates for GC, HGIN, LGIN and precancerous conditions were 2.36, 0.53, 1.66 and 37.94%, respectively. Notably, the Q4 group accounted for 62.25% of GC cases, 58.56% of HGIN cases, 40.76% of LGIN cases and 30.88% of precancerous conditions (Fig. 5a, Table 3). Across the six previously published models, the cumulative contribution of different risk groups to the detection of GC, GPL, and precancerous conditions varied substantially (Fig. 5b–g, Table 3).

**Table 3:** Detection rates and distribution of gastric lesions across risk score quartiles among gastric cancer risk assessment models

Models	Risk group	Number	Gastric cancer		Gastric precancerous lesions				Gastric precancerous conditions		Inflammation /Normal		Others	
					LGIN		HGIN							
			n.	%	n.	%	n.	%	n.	%	n.	%	n.	%
Eom (Male)	Q1	11,443	33	0.29	67	0.59	5	0.04	2,493	21.79	8,650	75.59	195	1.70
	Q2	11,595	93	0.80	109	0.94	17	0.15	3,676	31.70	7,510	64.77	190	1.64
	Q3	11,322	155	1.37	194	1.71	62	0.55	4,323	38.18	6,398	56.51	190	1.68
	Q4	11,404	371	3.25	255	2.24	81	0.71	4,895	42.92	5,573	48.87	229	2.01
	Total	45,764	652	1.42	625	1.37	165	0.36	15,387	33.62	28,131	61.47	804	1.76
Eom (Female)	Q1	12,897	111	0.86	134	1.04	28	0.22	4,192	32.50	7,998	62.01	434	3.37
	Q2	13,228	68	0.51	104	0.79	12	0.09	3,741	28.28	8,857	66.96	446	3.37
	Q3	12,875	55	0.43	82	0.64	8	0.06	3,663	28.45	8,596	66.77	471	3.66
	Q4	12,578	49	0.39	61	0.48	9	0.07	3,324	26.43	8,653	68.79	482	3.83
	Total	51,578	283	0.55	381	0.74	57	0.11	14,920	28.93	34,104	66.12	1,833	3.55
Oh	Q1	6,397	24	0.38	37	0.58	15	0.23	1,696	26.51	4,435	69.33	190	2.97
	Q2	6,397	19	0.30	45	0.70	8	0.13	2,100	32.83	4,002	62.56	223	3.49
	Q3	6,397	37	0.58	92	1.44	10	0.16	2,277	35.59	3,785	59.17	196	3.06
	Q4	6,397	44	0.69	133	2.08	21	0.33	3,431	53.63	2,622	40.99	146	2.28
	Total	25,588	124	0.48	307	1.20	54	0.21	9,504	37.14	14,844	58.01	755	2.95
Zhu	Q1	25,139	66	0.26	104	0.41	12	0.05	5,276	20.99	18,871	75.07	810	3.22
	Q2	23,897	113	0.47	166	0.69	20	0.08	6,980	29.21	15,899	66.53	719	3.01
	Q3	24,212	191	0.79	276	1.14	50	0.21	8,383	34.62	14,702	60.72	610	2.52
	Q4	24,094	565	2.34	460	1.91	140	0.58	9,668	40.13	12,763	52.97	498	2.07
	Total	97,342	935	0.96	1,006	1.03	222	0.23	30,307	31.13	62,235	63.93	2,637	2.71
Zhou	Q1	23,846	71	0.30	105	0.44	9	0.04	5,737	24.06	17,074	71.60	850	3.56
	Q2	29,590	161	0.54	255	0.86	31	0.10	9,254	31.27	19,081	64.48	808	2.73
	Q3	12,217	119	0.97	146	1.20	37	0.30	4,454	36.46	7,222	59.11	239	1.96
	Q4	15,825	324	2.05	340	2.15	96	0.61	6,406	40.48	8,351	52.77	308	1.95
	Total	81,478	675	0.83	846	1.04	173	0.21	25,851	31.73	51,728	63.49	2,205	2.71
Cao	Q1	42,352	191	0.45	304	0.72	65	0.15	11,049	26.09	29,557	69.79	1,186	2.80
	Q2	9,001	57	0.63	105	1.17	9	0.10	3,501	38.90	5,126	56.95	203	2.26
	Q3	26,123	245	0.94	277	1.06	63	0.24	8,035	30.76	16,820	64.39	683	2.61
	Q4	12,493	202	1.62	185	1.48	39	0.31	4,772	38.20	6,927	55.45	368	2.95
	Total	89,969	695	0.77	871	0.97	176	0.20	27,357	30.41	58,430	64.94	2,440	2.71
New model	Q1	22,437	46	0.21	141	0.63	19	0.08	6,059	27.00	15,407	68.67	765	3.41
	Q2	24,809	93	0.37	183	0.74	20	0.08	6,724	27.10	17,010	68.56	779	3.14
	Q3	25,428	214	0.84	272	1.07	53	0.21	8,166	32.11	16,101	63.32	622	2.45
	Q4	24,668	582	2.36	410	1.66	130	0.53	9,358	37.94	13,717	55.61	471	1.91
	Total	97,342	935	0.96	1,006	1.03	222	0.23	30,307	31.13	62,235	63.93	2,637	2.71

*HGIN* high-grade intraepithelial neoplasia, *LGIN* low-grade intraepithelial neoplasia

**Fig. 6:**
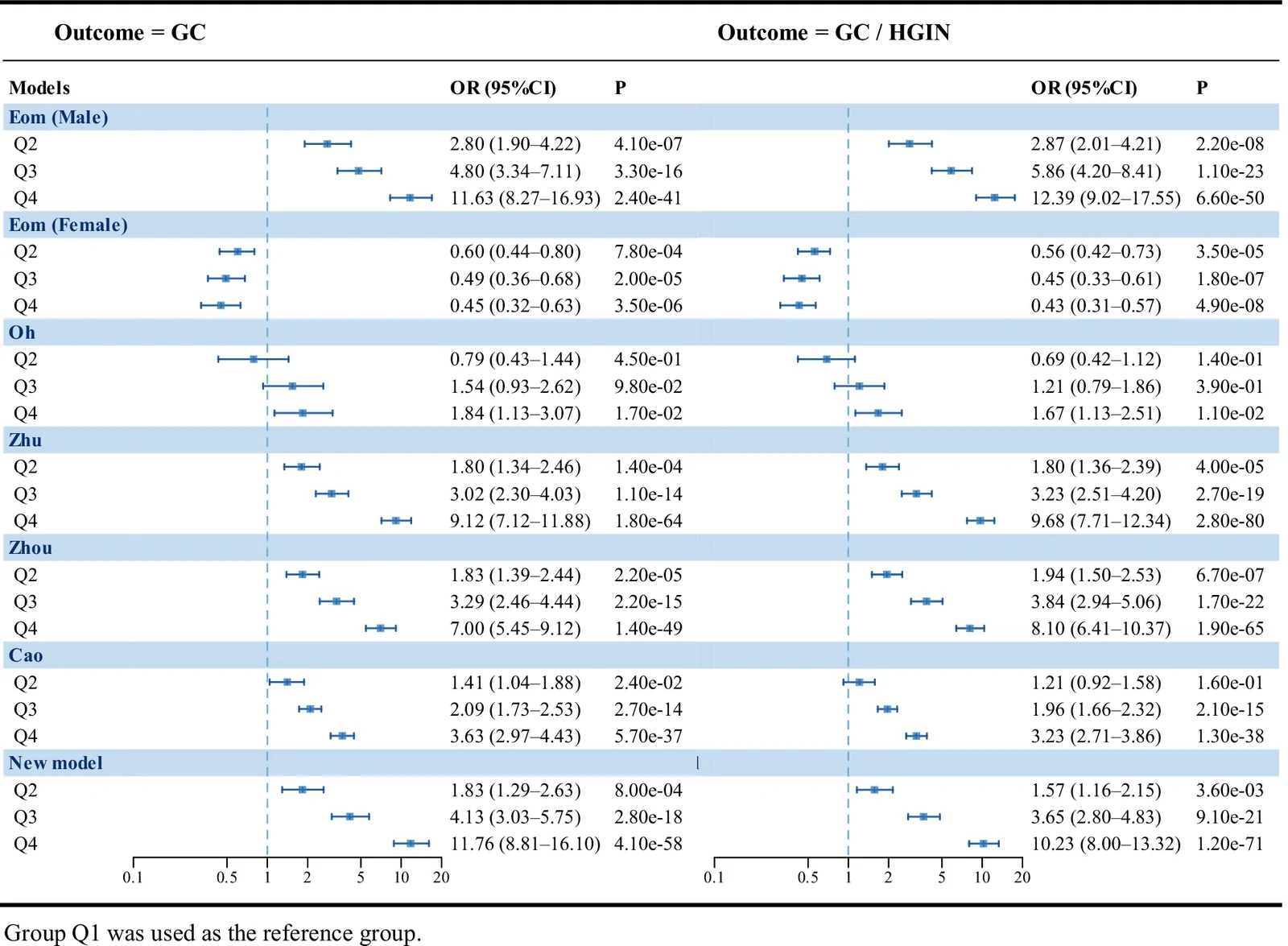
Odds ratios for GC and GC/HGIN across risk score quartiles in different models

Risk stratification analyses based on the odds ratios demonstrated a consistent gradient of increasing risk across quartiles for the new model, as well as in the Eom (Male) [9], Zhou [12], Zhu [13], Oh [10], and Cao [11] models. Compared with Q1, participants in Q2-Q4 showed progressively higher odds of both GC and GC/HGIN. In contrast, the Eom (Female) model [9] showed an inverse trend across quartiles, suggesting limited transportability and suboptimal performance in the present cohort (Fig. 6).

**Fig. 7:**
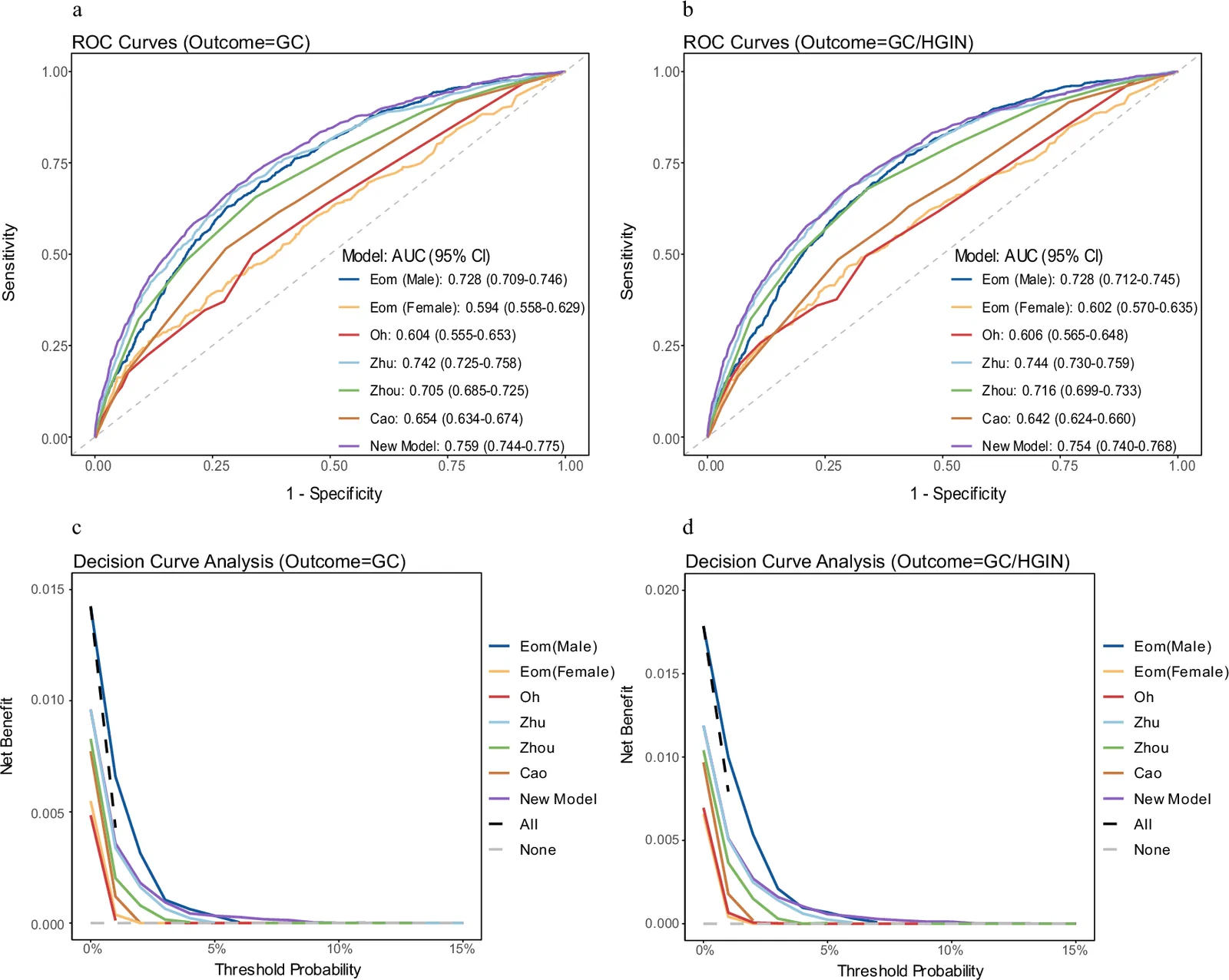
Comparisons of receiver operating characteristic (ROC) curves and decision curve analysis (DCA) among GC risk assessment models. **a**: ROC curves for GC; **b**: ROC curves for GC/HGIN; **c**: DCA for GC; **d**: DCA for GC/HGIN

ROC analysis showed that the new model achieved the highest discriminative performance for GC (AUC = 0.759, 95% CI: 0.744–0.775), significantly outperforming the Eom (Male) [9], Oh [10], Zhou [12], Cao [11], and Eom (Female) [9] models (Bootstrap test, all *P* < 0.05), while showing comparable performance to the Zhu model [13] (Bootstrap test, *P* = 0.128, Fig. 7a). Three models [9], Zhou [12], and Zhu [13]] achieved AUC values above 0.70, indicating acceptable discrimination, whereas the Eom (Female) [9], Oh [10], and Cao [11] models demonstrated relatively modest discriminative ability, with AUC values below 0.70 (Fig. 7a). Similar findings were observed when GC and HGIN were combined as the outcome, for which the new model also yielded the highest AUC of 0.754 (95% CI: 0.740–0.768), outperforming all comparator models (Fig. 7b).

Decision curve analysis demonstrated that both the new model and the Eom (Male) [9] model provided greater net benefit across a broad range of threshold probabilities for both GC and GC/HGIN outcomes, indicating improved potential clinical utility for risk stratification and screening decision-making in the target population. In comparison, the Zhu [13] and Zhou [12] models showed relatively limited net benefit, whereas the Eom (Female) [9], Oh [10], and Cao [11] models offered minimal clinical utility within the evaluated threshold ranges (Fig. 7c–d).

When Q4 was defined as the high-risk group, four models achieved PPVs above 2.00% for GC. The Eom (Male) model [9] yielded the highest PPV (3.25%), followed by the new model (2.36%), Zhu model [13] (2.34%), and Zhou model [12] (2.05%). For the composite outcome of GC/HGIN, the same four models achieved PPVs above 2.00%. When Q1-Q3 were defined as the non-high-risk group, NPVs ranged from 99.18% to 99.58% for GC and from 98.94% to 99.41% for GC/HGIN across the models (Table 4).

**Table 4:** Positive and negative predictive values of high-risk classification across gastric cancer risk assessment models

Model	PPV in high-risk group (Q4)	NPV in non-high-risk group (Q1-Q3)
Outcome = GC		
Eom (Male)	371/11404(3.25%)	34079/34360 (99.18%)
Eom (Female)	49/12578(0.39%)	38766/39000 (99.40%)
Oh	44/6397(0.69%)	19111/19191 (99.58%)
Zhu	565/24094(2.34%)	72878/73248 (99.49%)
Cao	202/12493(1.62%)	76983/77476 (99.36%)
Zhou	324/15825(2.05%)	65302/65653 (99.47%)
New Model	582/24668(2.36%)	72321/72674 (99.51%)
Outcome = GC/HGIN		
Eom (Male)	452/11404(3.96%)	33995/34360 (98.94%)
Eom (Female)	58/12578(0.46%)	38718/39000 (99.28%)
Oh	65/6397(1.02%)	19078/19191 (99.41%)
Zhu	705/24094(2.93%)	72796/73248 (99.38%)
Cao	241/12493(1.93%)	76846/77476 (99.19%)
Zhou	420/15825(2.65%)	65225/65653 (99.35%)
New Model	712/24668(2.89%)	72229/72674 (99.39%)

*PPV* positive predictive value, *NPV* negative predictive value, *GC* gastric cancer, *HGIN* high-grade intraepithelial neoplasia

## Discussion

This study, based on a large-scale multicenter endoscopic screening cohort in Zhejiang Province, China, developed and validated a novel gastric cancer risk assessment model incorporating *H. pylori* infection status and systematically evaluated the applicability of six previously published models in a large Chinese population. Overall, the newly developed model demonstrated favorable discrimination, clinical utility, and geographic robustness across different regional subsets, supporting its potential applicability for risk-stratified gastric cancer screening in multicenter populations.

The newly developed model incorporated 9 readily obtainable demographic, lifestyle, dietary, and clinical variables, providing a practical tool for large-scale GC screening. In addition to demonstrating stable discriminatory performance in the validation cohort, the model also maintained relatively consistent performance across different geographic regions of Zhejiang Province, suggesting potential robustness in multicenter screening settings. Furthermore, the high-risk group identified by the model effectively concentrated GC, HGIN, LGIN and precancerous condition cases, supporting the model’s utility for identifying individuals who may benefit most from targeted endoscopic screening.

Previous studies have demonstrated that *H. pylori* infection plays a central role in gastric carcinogenesis and that eradication therapy can substantially reduce GC risk [14]. Given the high burden of *H. pylori* infection and gastric cancer in China, incorporation of *H. pylori* testing into population-based screening strategies may improve risk stratification and screening efficiency [15]. Compared with previously published models, the inclusion of *H. pylori* infection status in our model may enhance its applicability and clinical relevance within the Chinese screening population.

Among the previously published models, those developed from Chinese populations appeared to demonstrate better applicability within the Zhejiang screening cohort, whereas the performance of models developed in other populations may have been influenced by differences in demographic characteristics, baseline risk profiles, and variable definitions. In addition, some published models were originally designed to predict future incident GC rather than lesions detected during endoscopic screening, and several required variables had to be approximated because of differences in data availability. These factors may have affected the transportability and comparative performance of the external models in the current screening setting. Notably, the Eom (Female) [9] model demonstrated an inverse risk stratification pattern in the present cohort. Several factors may explain this finding. First, the original Korean female model incorporated a strong quadratic age term, with predicted risk peaking around the mid-50s and subsequently declining at older ages. In contrast, the current Chinese screening cohort included a substantially older age distribution, with a large proportion of participants aged 60 years or above, among whom GC cases were more concentrated. By comparison, the age-related risk pattern in the Eom (Male) [9] model remained relatively stable and continued to increase across the age range represented in the present cohort, which may partly explain its better transportability and discriminative performance. Therefore, the present findings should be interpreted as an evaluation of model applicability within the current screening context rather than a definitive comparison of model superiority. Nevertheless, the newly developed model demonstrated relatively stable performance across multiple analytical settings, supporting its potential utility in multicenter screening populations.

If gastric cancer is diagnosed at an early stage, the 5-year survival rate can approach 95–99%; however, survival remains substantially lower in advanced-stage disease [16–19], underscoring the importance of early detection. Japan and Korea have established nationwide gastric cancer screening systems centered on endoscopic examination, which have substantially improved early detection and survival outcomes [20]. In China, several regional and national upper gastrointestinal cancer screening programs have gradually expanded since 2005 [21, 22]. Although screening coverage and early detection have improved, China still faces substantial challenges in implementing population-wide endoscopic screening, including limited healthcare resources, uneven distribution of endoscopic capacity, and variable screening quality.

Against this background, risk-stratified screening strategies may provide a more practical and resource-efficient approach for large-scale GC prevention in China. Similar to screening strategies proposed in Japan [23], our findings support a stepwise screening framework in which questionnaire-based risk assessment and *H. pylori* testing are used to identify individuals at elevated risk before targeted endoscopic examination. Such an approach may improve screening efficiency and optimize allocation of limited endoscopic resources.

From a practical implementation perspective, the selection of an appropriate risk-score threshold for defining the high-risk population should balance sensitivity, specificity, and endoscopic resource availability. Lower cut-off values may improve the detection of gastric cancer and clinically significant neoplastic lesions but could substantially increase the endoscopic workload and healthcare burden. In contrast, higher cut-off values may improve screening efficiency and reduce unnecessary endoscopic examinations but may also increase the risk of missed lesions. Therefore, the optimal threshold may vary according to local healthcare resources, screening priorities, population risk profiles, and cost-effectiveness considerations in different screening settings.

This study has several strengths and innovations. First, it was based on more than 140,000 participants from a large-scale multicenter endoscopic screening cohort in Zhejiang Province, making it one of the largest cohorts for gastric cancer risk assessment in China and enhancing the representativeness and robustness of our findings. Second, the new model was constructed using easily obtainable questionnaire-based variables combined with *H. pylori* infection status, making it highly feasible for application in routine screening and primary healthcare settings. Third, beyond model development, this study systematically validated and compared six previously published models, providing a comprehensive evaluation of their applicability in a real-world Chinese screening population.

Several limitations should also be acknowledged. First, the cross-sectional nature of the screening data limited causal inference and precluded assessment of long-term predictive ability. Second, although the model demonstrated relatively stable performance across different regional subsets within Zhejiang Province, all participants were derived from the same provincial screening network. Therefore, the generalizability and transportability of the model to other geographic regions, populations, and healthcare settings should be interpreted cautiously and require further independent external validation. Third, the model relied primarily on self-reported questionnaire data and single-point measurements, potentially raising the possibility of information bias. In addition, some discrepancies in variable definitions between our dataset and the previously published models may have influenced validation performance. Fourth, biomarker-based prediction models could not be evaluated because several blood-based biomarkers were unavailable in the current screening dataset. This limitation prevents a comprehensive comparison across the full spectrum of existing models and highlights an important avenue for future research.

## Conclusions

 In conclusion, the newly developed model demonstrated favorable discrimination and effective risk stratification, with relatively consistent performance across geographic subregions within Zhejiang Province, supporting its potential utility for risk-adapted gastric cancer screening. Future studies should perform independent external validation in broader geographic regions and populations to further assess the generalizability and transportability of the model. In addition, incorporation of serological, genetic, and other emerging risk factors may further improve risk assessment performance. Exploring implementation within community-based screening systems may facilitate precision, risk-stratified gastric cancer prevention and more efficient allocation of healthcare resources.

## Supplementary information

Supplementary Material 1: Additional file: Tables S1–S4.

## Data Availability

The datasets generated and analyzed during the current study are not publicly available because they contain potentially identifiable personal health information and are subject to institutional ethical and data protection restrictions. De-identified data may be made available from the corresponding author upon reasonable request for academic research purposes, subject to approval by the institutional ethics committee and completion of a detailed re-analysis plan and a data use agreement. Requests for data access will typically be reviewed within 4 weeks. Data recipients are not permitted to redistribute the data and must comply with the conditions specified in the approved data use agreement and applicable ethical regulations.The R codes used for model development, external validation, and statistical analyses are publicly available at: https://github.com/Wendy-ChenYing/GC-risk-model.

## Abbreviations

AJCC: American Joint Committee on Cancer
AUC: Area Under the Curve
BMI: Body Mass Index
CI: Confidence Interval
DCA: Decision Curve Analysis
GC: Gastric Cancer
GPL: Gastric Precancerous Lesions
LASSO: Least absolute shrinkage and selection operator
LGIN: Low-grade intraepithelial neoplasia
HGIN: High-grade intraepithelial neoplasia
*H. pylori*: *Helicobacter pylori*
NPV: Negative Predictive Value
OR: Odds Ratio
PPV: Positive Predictive Value
ROC: Receiver Operating Characteristic
SD: Standard Deviation
