# Supplementary material for: Comparative validation and local adaptation of gastric cancer risk assessment models in a large multicenter endoscopic screening cohort in China
Source: BMC Med. 2026 Jul 24;24:577. doi: 10.1186/s12916-026-05082-6 (PMC13685247; doi:10.1186/s12916-026-05082-6)
Supplement: Supplementary file 1 [file 12916_2026_5082_MOESM1_ESM.docx]

**Additional file: Table S1- Table S4**

Table S1. Characteristics of Six Previously Published Gastric Cancer Risk Assessment Models.

Table S2. Definitions, Harmonization, and Availability of Variables Included in the Gastric Cancer Risk Assessment Models.

Table S3. Baseline Characteristics of Participants in the Training and Validation Sets.

Table S4. Univariable Logistic Regression Analysis of Gastric Cancer Risk Factors in the Training Set.

| Model | | Country | Age (years) | Study design | Model type | Sample size | Included risk factors | Discrimination of original included models |
| --- | --- | --- | --- | --- | --- | --- | --- | --- |
| Eom BW et al (2015) | Male model | Korea | 30-80 | Cohort | Cox | 1,372,424 (development) and 484,335 (validation) | Age, BMI, Family history of cancer, Meal regularity, Salt preference, Alcohol consumption, Smoking amount, Physical activity | Development AUC:0.764(0.760-0.768)  Validation AUC:0.782(0.777-0.787) |
|  | Female model | Korea | 30-80 | Cohort | Cox | 804,077 (development) and 466,013(validation) | Age, BMI, Family history of cancer, Salt preference, Alcohol consumption, Smoking | Development AUC:0.706(0.698-0.715)  Validation AUC:0.705(0.696-0.714) |
| Oh H et al (2024) | | Korea | ≥20 | Cohort | Cox | 260,407(full cohort) | Age, Sex, Smoking status, Alcohol consumption, Family history of cancer, Previous results for endoscopy | Development AUC:0.800(0.770-0.829)  Validation AUC:0.799(0.743-0.856) |
| Cao M et al (2020) | | China | 40-69 | Cluster-randomized controlled trial | Cox | 48,295(screening) and 41,619(control) | Smoking, Drinking, Diet habit, Family history of upper gastrointestinal cancer, History of gastric disease | / |
| Zhou R et al (2021) | | China | 45-69 | Cohort | Gastric cancer risk scoring system | 8,214 and 7,235 (two outpatient cohorts); 32,630(community-based cohort) | Age, Sex, Family history of gastric cancer, smoking, Drinking, BMI, Consumption of salty food, Meal irregularity, Consumption of preserved food | Validation AUC:0.763(0.707-0.819); 0.706(0.606-0.807); 0.696(0.567-0.825) |
| Zhu X et al (2023) | | China | 40-75 | Cohort | Cox | 416,343(development);13,982 and 5,348(two validation cohorts) | Age, Sex, Education, BMI, Pack-years of smoking, Alcohol drinking, Intake of fresh vegetables and fruits, Intake of salty foods, Previous cancer diagnosis, Family history of cancer in first-degree relatives, History of peptic ulcer | Development AUC:0.754(0.745-0.762)  Validation AUC:0.736(0.710-0.761) |

Table S1. Characteristics of Six Previously Published Gastric Cancer Risk Assessment Models

Table S2. Definitions, Harmonization, and Availability of Variables Included in the Gastric Cancer Risk Assessment Models

| Model | The variables definition of the original model | | The definition of variables in the new validation set |
| --- | --- | --- | --- |
| Eom BW et al (2015) (Male model) | BMI(<18.5kg/m^2^;18.5-22.9 kg/m^2^;23.0-24.9kg/m^2^; ≥25.0 kg/m^2^) | Consistent with the original model | |
|  | Family history of cancer (Yes; No) | Classification based on the family history of gastric cancer in first-degree relatives. | |
|  | Meal regularity (Regular; Intermediate; Irregular) | In the absence of this variable, it defaults to " Regular ". | |
|  | Salt preference (Not salty; Intermediate; Salty) | In the absence of this variable, it defaults to " Not salty ". | |
|  | Alcohol consumption (None; 1–14.9 g/day; 15–24.9 g/day; 25 more g/day) | Consistent with the original model | |
|  | Smoking amount (None Smoker; Past Smoker; Current Smoker, <0.5 pack/day; Current Smoker, 0.5–0.99 pack/day; Current Smoker, ≥1 pack/day) | The "Past smoker" variable is absent; the classification of the remaining variables is consistent with the original model. | |
|  | Physical Activity (None, Light, Moderate to high) | In the absence of this variable, it defaults to " None ". | |
| Eom BW et al (2015) (Female model) | BMI(<18.5kg/m^2^;18.5-22.9 kg/m^2^;23.0-24.9kg/m^2^;≥25.0 kg/m^2^) | Consistent with the original model | |
|  | Family history of cancer (Yes; No) | Classification based on the family history of gastric cancer in first-degree relatives. | |
|  | Salt preference (Not salty; Intermediate; Salty) | In the absence of this variable, it defaults to " Not salty ". | |
|  | Alcohol consumption (None; 1–14.9 g/day; 15 more g/day) | Consistent with the original model | |
|  | Smoking [None Smoker; Smoker (Past, Current)] | The "Past smoker" variable is absent; the classification of the remaining variables is consistent with the original model. | |
| Oh H et al (2024) | Age (≥20 years, with a classification interval of 10 years.) | Consistent with the original model | |
|  | Sex (Men; Women) | Consistent with the original model | |
|  | Alcohol consumption (<2 times/week; ≥2 times/week) | Consistent with the original model | |
|  | Smoking status (Never smoker; Ever smoker) | Consistent with the original model | |
|  | Family history of cancer (No; Family history of gastric cancer; Family history of other cancer) | Classification based on the family history of gastric cancer in first-degree relatives. | |
|  | Endoscopic results (No; Atrophic gastritis; Intestinal metaplasia) | The endoscopic diagnoses of esophageal low-grade intraepithelial neoplasia, Barrett's esophagus, and gastric low-grade intraepithelial neoplasia, along with intestinal metaplasia, were classified as same group, while chronic gastritis, duodenal ulcer, and postoperative benign gastric remnants were classified as the "No" group. | |
| Cao M et al (2020) | Smoking (≥20 cigarettes/day, ≥10 years) | Consistent with the original model | |
|  | Alcohol consumption (≥28g/day, ≥10 years) | Consistent with the original model | |
|  | Pickled food (≥1 time/week) | Set as “1” for ≥2 times/week, and “0” for <2 times/week | |
|  | Eating habits (Hot food: ≥70℃; Hard food: indigestible food) | The " hard food " variable is absent; the classification of the remaining variables is consistent with the original model. | |
|  | Family history of upper gastrointestinal cancer (Yes; No) | Consistent with the original model | |
|  | Chest pain/oppression/burning sensation (Yes; No) | Consistent with the original model | |
|  | Difficulty swallowing (Yes; No) | Consistent with the original model | |
|  | Chronic gastric burning/indigestion (Yes; No) | The " chronic gastric burning " variable is absent; the classification of the remaining variables is consistent with the original model. | |
|  | Vomiting/hematemesis (Yes; No) | Grouped according to vomiting, hematemesis variable is absent | |
|  | Progressive weight loss (Yes; No) | In the absence of this variable, it defaults to " No ". | |
|  | Esophageal reflux (Yes; No) | Consistent with the original model | |
|  | Gastric ulcer, superficial gastritis, atrophic gastritis (Yes; No) | The " superficial gastritis " variable is absent; the classification of the remaining variables is consistent with the original model. | |
| Zhou R et al (2021) | Age (40-69 years old, with a classification interval of 5 years) | Consistent with the original model | |
|  | Sex (Men; Women) | Consistent with the original model | |
|  | Family history of gastric cancer (Immediate relatives or collateral relatives within three generations, Yes; No) | Classification based on the family history of gastric cancer in first-degree relatives. | |
|  | Smoking (<18 packs/year; ≥18 packs/year) | Consistent with the original model | |
|  | Alcohol consumption (Alcohol content >40%; ≥twice a week; lasting ≥12 months, Yes; No) | Consistent with the original model | |
|  | BMI (<18.5kg/m^2^; ≥18.5 kg/m^2^) | Consistent with the original model | |
|  | Salt preference (<1 time/week; ≥1 time/week salty food) | In the absence of this variable, it defaults to "<1 time/week ". | |
|  | Meal regularity (Yes; No) | In the absence of this variable, it defaults to "No ". | |
|  | Pickled food (<1 time/week; ≥1 time/week) | Set as “0” for <2 times/week, and “1” for ≥2 times/week | |
| Zhu X et al (2023) | Age (40-75 years old, with a classification interval of 5 years) | Consistent with the original model | |
|  | Sex (Men; Women) | Consistent with the original model | |
|  | Education level (Primary school and below; Junior high school; Senior high school; College and above) | Consistent with the original model | |
|  | BMI (<18.5kg/m^2^; ≥18.5 kg/m^2^) | Consistent with the original model | |
|  | Smoking (0; <20 pack-years; ≥20 pack-years) | Consistent with the original model | |
|  | Alcohol consumption (The boundary value is 15g/day for females and 25g/day for males in terms of daily alcohol consumption) | Consistent with the original model | |
|  | Intake of vegetables and fruits (Frequently: intake of vegetables every day and intake of fruits ≥4 days/week, or intake of fruits every day and intake of vegetables ≥4 days/week; the rest are grouped as “Occasionally”) | Consistent with the original model | |
|  | Pickled food (Frequently: ≥4 days/week; Occasionally: <4 days/week) | Consistent with the original model | |
|  | Past cancer history (Yes; No) | In the absence of this variable, it defaults to "No". | |
|  | Family history of cancer in first-degree relatives (Yes; No) | Consistent with the original model | |
|  | History of peptic ulcer (Yes; No) | Consistent with the original model | |

Table S3. Baseline Characteristics of Participants in the Training and Validation Sets

|  | Training set | | | | | Validation set | | | | |
| --- | --- | --- | --- | --- | --- | --- | --- | --- | --- | --- |
| Characteristics | All  n= 46,987 | Non-gastric cancer  n = 46,503 | Gastric cancer  n = 484 | *χ^2^*value | *P* value | All  n = 97,342 | Non-gastric cancer  n = 96,407 | Gastric cancer  n = 935 | *χ^2^*value | *P* value |
| Age (years) (n, %) |  |  |  | 274 | <0.001 |  |  |  | 617 | <0.001 |
| 40-49 | 11,026 (23.47%) | 10,987 (23.63%) | 39 (8.06%) |  |  | 20,683 (21.25%) | 20,626 (21.39%) | 57 (6.10%) |  |  |
| 50-59 | 19,296 (41.07%) | 19,176 (41.24%) | 120 (24.79%) |  |  | 38,871 (39.93%) | 38,644 (40.08%) | 227 (24.28%) |  |  |
| 60-69 | 13,319 (28.35%) | 13,098 (28.17%) | 221 (45.66%) |  |  | 30,415 (31.25%) | 30,004 (31.12%) | 411 (43.96%) |  |  |
| 70-74 | 3,346 (7.12%) | 3,242 (6.97%) | 104 (21.49%) |  |  | 7,373 (7.57%) | 7,133 (7.40%) | 240 (25.67%) |  |  |
| Sex (n, %) |  |  |  | 92.0 | <0.001 |  |  |  | 195 | <0.001 |
| Female | 24,881 (52.95%) | 24,730 (53.18%) | 151 (31.20%) |  |  | 51,578 (52.99%) | 51,295 (53.21%) | 283 (30.27%) |  |  |
| Male | 22,106 (47.05%) | 21,773 (46.82%) | 333 (68.80%) |  |  | 45,764 (47.01%) | 45,112 (46.79%) | 652 (69.73%) |  |  |
| Education (n, %) |  |  |  | 80.2 | <0.001 |  |  |  | 116 | <0.001 |
| primary school education or below | 19,290 (41.05%) | 19,002 (40.86%) | 288 (59.50%) |  |  | 40,740 (41.85%) | 40,209 (41.71%) | 531 (56.79%) |  |  |
| junior high school | 15,586 (33.17%) | 15,453 (33.23%) | 133 (27.48%) |  |  | 32,107 (32.98%) | 31,820 (33.01%) | 287 (30.70%) |  |  |
| senior high school | 7,338 (15.62%) | 7,289 (15.67%) | 49 (10.12%) |  |  | 14,218 (14.61%) | 14,134 (14.66%) | 84 (8.98%) |  |  |
| junior college education or above | 4,773 (10.16%) | 4,759 (10.23%) | 14 (2.89%) |  |  | 10,277 (10.56%) | 10,244 (10.63%) | 33 (3.53%) |  |  |
| BMI, kg/m^2^ (n, %) |  |  |  | 8.41 | 0.004 |  |  |  | 24.9 | <0.001 |
| ＜18.5 | 1,279 (2.72%) | 1,255 (2.70%) | 24 (4.96%) |  |  | 2,392 (2.46%) | 2,345 (2.43%) | 47 (5.03%) |  |  |
| ≥18.5 | 45,708 (97.28%) | 45,248 (97.30%) | 460 (95.04%) |  |  | 94,950 (97.54%) | 94,062 (97.57%) | 888 (94.97%) |  |  |
| Smoking (n, %) |  |  |  | 70.8 | <0.001 |  |  |  | 121 | <0.001 |
| No | 38,555 (82.05%) | 38,229 (82.21%) | 326 (67.36%) |  |  | 80,794 (83.00%) | 80,144 (83.13%) | 650 (69.52%) |  |  |
| Yes | 8,432 (17.95%) | 8,274 (17.79%) | 158 (32.64%) |  |  | 16,548 (17.00%) | 16,263 (16.87%) | 285 (30.48%) |  |  |
| Drinking (n, %) |  |  |  | 32.5 | <0.001 |  |  |  | 89.1 | <0.001 |
| No | 38,573 (82.09%) | 38,224 (82.20%) | 349 (72.11%) |  |  | 82,686 (84.94%) | 81,995 (85.05%) | 691 (73.90%) |  |  |
| Yes | 8,414 (17.91%) | 8,279 (17.80%) | 135 (27.89%) |  |  | 14,656 (15.06%) | 14,412 (14.95%) | 244 (26.10%) |  |  |
| Intake of pickled food (n, %) |  |  |  | 5.06 | 0.024 |  |  |  | 97.1 | <0.001 |
| ≤3 days/week | 37,950 (80.77%) | 37,579 (80.81%) | 371 (76.65%) |  |  | 81,931 (84.17%) | 81,254 (84.28%) | 677 (72.41%) |  |  |
| ＞3 days/week | 9,037 (19.23%) | 8,924 (19.19%) | 113 (23.35%) |  |  | 15,411 (15.83%) | 15,153 (15.72%) | 258 (27.59%) |  |  |
| Intake of vegetables (n, %) |  |  |  | 78.2 | <0.001 |  |  |  | 0.04 | 0.8 |
| ≤3 days/week | 6,894 (14.67%) | 6,754 (14.52%) | 140 (28.93%) |  |  | 16,172 (16.61%) | 16,014 (16.61%) | 158 (16.90%) |  |  |
| ＞3 days/week | 40,093 (85.33%) | 39,749 (85.48%) | 344 (71.07%) |  |  | 81,170 (83.39%) | 80,393 (83.39%) | 777 (83.10%) |  |  |
| Intake of fruits (n, %) |  |  |  | 74.1 | <0.001 |  |  |  | 33.3 | <0.001 |
| ≤3 days/week | 18,581 (39.54%) | 18,297 (39.35%) | 284 (58.68%) |  |  | 41,906 (43.05%) | 41,416 (42.96%) | 490 (52.41%) |  |  |
| ＞3 days/week | 28,406 (60.46%) | 28,206 (60.65%) | 200 (41.32%) |  |  | 55,436 (56.95%) | 54,991 (57.04%) | 445 (47.59%) |  |  |
| Intake of hot food/tea (n, %) |  |  |  | 10.1 | 0.002 |  |  |  | 49.6 | <0.001 |
| No | 36,273 (77.20%) | 35,929 (77.26%) | 344 (71.07%) |  |  | 79,392 (81.56%) | 78,713 (81.65%) | 679 (72.62%) |  |  |
| Yes | 10,714 (22.80%) | 10,574 (22.74%) | 140 (28.93%) |  |  | 17,950 (18.44%) | 17,694 (18.35%) | 256 (27.38%) |  |  |
| History of gastroscopy (n, %) |  |  |  | 75.8 | <0.001 |  |  |  | 82.0 | <0.001 |
| No | 33,885 (72.12%) | 33,450 (71.93%) | 435 (89.88%) |  |  | 71,754 (73.71%) | 70,943 (73.59%) | 811 (86.74%) |  |  |
| Yes | 13,102 (27.88%) | 13,053 (28.07%) | 49 (10.12%) |  |  | 25,588 (26.29%) | 25,464 (26.41%) | 124 (13.26%) |  |  |
| History of peptic ulcer (n, %) |  |  |  | 2.14 | 0.14 |  |  |  | 1.05 | 0.3 |
| No | 44,494 (94.69%) | 44,028 (94.68%) | 466 (96.28%) |  |  | 94,409 (96.99%) | 93,508 (96.99%) | 901 (96.36%) |  |  |
| Yes | 2,493 (5.31%) | 2,475 (5.32%) | 18 (3.72%) |  |  | 2,933 (3.01%) | 2,899 (3.01%) | 34 (3.64%) |  |  |
| Upper gastrointestinal symptoms (n, %) |  |  |  | 67.1 | <0.001 |  |  |  | 141 | <0.001 |
| No | 25,419 (54.10%) | 25,247 (54.29%) | 172 (35.54%) |  |  | 56,287 (57.82%) | 55,925 (58.01%) | 362 (38.72%) |  |  |
| Yes | 21,568 (45.90%) | 21,256 (45.71%) | 312 (64.46%) |  |  | 41,055 (42.18%) | 40,482 (41.99%) | 573 (61.28%) |  |  |
| Family history of gastric cancer in first-degree relatives (n, %) |  |  |  | 0.50 | 0.5 |  |  |  | 0.05 | 0.8 |
| No | 46,337 (98.62%) | 45,862 (98.62%) | 475 (98.14%) |  |  | 95,714 (98.33%) | 94,796 (98.33%) | 918 (98.18%) |  |  |
| Yes | 650 (1.38%) | 641 (1.38%) | 9 (1.86%) |  |  | 1,628 (1.67%) | 1,611 (1.67%) | 17 (1.82%) |  |  |
| *H. pylori* infection status (n, %) |  |  |  | 44.6 | <0.001 |  |  |  | 41.3 | <0.001 |
| Positive | 13,951 (29.69%) | 13,740 (29.55%) | 211 (43.60%) |  |  | 25,924 (26.63%) | 25,588 (26.54%) | 336 (35.94%) |  |  |
| Negative | 33,036 (70.31%) | 32,763 (70.45%) | 273 (56.40%) |  |  | 71,418 (73.37%) | 70,819 (73.46%) | 599 (64.06%) |  |  |

BMI, body mass index; *H. pylori*, *Helicobacter pylori*.

Table S4. Univariable Logistic Regression Analysis of Gastric Cancer Risk Factors in the Training Set

| Characteristics | Non-gastric cancer  (n=46,503) | Gastric cancer  (n=484) | OR | 95%CI | *P* value |
| --- | --- | --- | --- | --- | --- |
| Age (years) (n, %) |  |  |  |  |  |
| 40-49 | 10987 (23.6%) | 39 (8.1%) | ref |  |  |
| 50-59 | 19176 (41.2%) | 120 (24.8%) | 1.76 | 1.23-2.53 | 0.002 |
| 60-69 | 13098 (28.2%) | 221 (45.7%) | 4.75 | 3.38-6.69 | <0.001 |
| 70-74 | 3242 (7.0%) | 104 (21.5%) | 9.04 | 6.24-13.08 | <0.001 |
| Sex (n, %) |  |  |  |  |  |
| Female | 24730 (53.2%) | 151 (31.2%) | ref |  |  |
| Male | 21773 (46.8%) | 333 (68.8%) | 2.50 | 2.06-3.04 | <0.001 |
| Education (n, %) |  |  |  |  |  |
| junior college education or above | 4759 (10.2%) | 14 (2.9%) | ref |  |  |
| senior high school | 7289 (15.7%) | 49 (10.1%) | 2.29 | 1.26-4.14 | 0.006 |
| junior high school | 15453 (33.2%) | 133 (27.5%) | 2.93 | 1.69-5.08 | <0.001 |
| primary school education or below | 19002 (40.9%) | 288 (59.5%) | 5.15 | 3.01-8.82 | <0.001 |
| BMI, kg/m^2^ (n, %) |  |  |  |  |  |
| ≥18.5 | 45248 (97.3%) | 460 (95.0%) | ref |  |  |
| ＜18.5 | 1255 (2.7%) | 24 (5.0%) | 1.88 | 1.24-2.85 | 0.003 |
| Smoking (n, %) |  |  |  |  |  |
| No | 38229 (82.2%) | 326 (67.4%) | ref |  |  |
| Yes | 8274 (17.8%) | 158 (32.6%) | 2.24 | 1.85-2.71 | <0.001 |
| Drinking (n, %) |  |  |  |  |  |
| No | 38224 (82.2%) | 349 (72.1%) | ref |  |  |
| Yes | 8279 (17.8%) | 135 (27.9%) | 1.79 | 1.46-2.18 | <0.001 |
| Intake of pickled food (n, %) |  |  |  |  |  |
| ≤3 days/week | 37579 (80.8%) | 371 (76.7%) | ref |  |  |
| ＞3 days/week | 8924 (19.2%) | 113 (23.3%) | 1.28 | 1.04-1.59 | 0.021 |
| Intake of vegetables (n, %) |  |  |  |  |  |
| ≤3 days/week | 6754 (14.5%) | 140 (28.9%) | ref |  |  |
| ＞3 days/week | 39749 (85.5%) | 344 (71.1%) | 2.40 | 1.96-2.92 | <0.001 |
| Intake of fruits (n, %) |  |  |  |  |  |
| ≤3 days/week | 18297 (39.3%) | 284 (58.7%) | ref |  |  |
| ＞3 days/week | 28206 (60.7%) | 200 (41.3%) | 2.19 | 1.83-2.63 | <0.001 |
| Intake of hot food/tea (n, %) |  |  |  |  |  |
| No | 35929 (77.3%) | 344 (71.1%) | ref |  |  |
| Yes | 10574 (22.7%) | 140 (28.9%) | 1.38 | 1.13-1.69 | 0.001 |
| History of gastroscopy (n, %) |  |  |  |  |  |
| Yes | 13053 (28.1%) | 49 (10.1%) | ref |  |  |
| No | 33450 (71.9%) | 435 (89.9%) | 3.46 | 2.58-4.66 | <0.001 |
| History of peptic ulcer (n, %) |  |  |  |  |  |
| No | 44028 (94.7%) | 466 (96.3%) | ref |  |  |
| Yes | 2475 (5.3%) | 18 (3.7%) | 0.69 | 0.43-1.10 | 0.120 |
| Upper gastrointestinal symptoms (n, %) |  |  |  |  |  |
| No | 25247 (54.3%) | 172 (35.5%) | ref |  |  |
| Yes | 21256 (45.7%) | 312 (64.5%) | 2.15 | 1.79-2.60 | <0.001 |
| Family history of gastric cancer in first-degree relatives (n, %) |  |  |  |  |  |
| No | 45862 (98.6%) | 475 (98.1%) | ref |  |  |
| Yes | 641 (1.4%) | 9 (1.9%) | 1.36 | 0.70-2.63 | 0.369 |
| *H. pylori* infection status (n, %) |  |  |  |  |  |
| Negative | 32763 (70.5%) | 273 (56.4%) | ref |  |  |
| Positive | 13740 (29.5%) | 211 (43.6%) | 1.84 | 1.54-2.21 | <0.001 |

BMI, body mass index; CI, confidence interval; *H. pylori*, *Helicobacter pylori*; OR, odds ratio.
